# Immune-modulatory Properties of the Octapeptide NAP in *Campylobacter jejuni* Infected Mice Suffering from Acute Enterocolitis

**DOI:** 10.3390/microorganisms8060802

**Published:** 2020-05-26

**Authors:** Markus M. Heimesaat, Soraya Mousavi, Sigri Kløve, Claudia Genger, Dennis Weschka, Eliezer Giladi, Stefan Bereswill, Illana Gozes

**Affiliations:** 1 Institute of Microbiology, Infectious Diseases and Immunology, Charité-University Medicine Berlin, Corporate Member of Freie Universität Berlin, Humboldt-Universität zu Berlin, and Berlin Institute of Health, 12203 Berlin, Germany; soraya.mousavi@charite.de (S.M.); sigri.klove@charite.de (S.K.); claudia.genger@charite.de (C.G.); dennis.weschka@charite.de (D.W.); stefan.bereswill@charite.de (S.B.); 2 Department of Human Molecular Genetics and Biochemistry, Sackler Faculty of Medicine, Sagol School of Neuroscience and Adams Super Center for Brain Studies, Tel Aviv University, Tel Aviv 69978, Israel; elieze@tauex.tau.ac.il (E.G.); igozes@tauex.tau.ac.il (I.G.)

**Keywords:** NAP, activity-dependent neuroprotective protein (ADNP), anti-inflammatory effects, immune-modulatory properties, *Campylobacter jejuni*, acute campylobacteriosis model, host–pathogen interaction, gut–brain axis

## Abstract

Human infections with the food-borne zoonotic pathogen *Campylobacter jejuni* are progressively rising and constitute serious global public health and socioeconomic burdens. Hence, application of compounds with disease-alleviating properties are required to combat campylobacteriosis and post-infectious sequelae. In our preclinical intervention study applying an acute *C. jejuni* induced enterocolitis model, we surveyed the anti-pathogenic and immune-modulatory effects of the octapeptide NAP which is well-known for its neuroprotective and anti-inflammatory properties. Therefore, secondary abiotic IL-10^−/−^ mice were perorally infected with *C. jejuni* and intraperitoneally treated with synthetic NAP from day 2 until day 5 post-infection. NAP-treatment did not affect gastrointestinal *C. jejuni* colonization but could alleviate clinical signs of infection that was accompanied by less pronounced apoptosis of colonic epithelial cells and enhancement of cell regenerative measures on day 6 post-infection. Moreover, NAP-treatment resulted in less distinct innate and adaptive pro-inflammatory immune responses that were not restricted to the intestinal tract but could also be observed in extra-intestinal and even systemic compartments. NAP-treatment further resulted in less frequent translocation of viable pathogens from the intestinal tract to extra-intestinal including systemic tissue sites. For the first time, we here provide evidence that NAP application constitutes a promising option to combat acute campylobacteriosis.

## 1. Introduction

Human campylobacteriosis cases that are mainly caused by the zoonotric pathogens *Campylobacter jejuni* and *C. coli* are progressively rising worldwide [1,2]. According to the European Centre for Disease Prevention and Control (ECDC), *C. jejuni* has become the most commonly reported infectious agents of bacterial gastroenteritis in the European Union (EU) since 2005 outcompeting *Salmonella*, *Yersinia*, and pathogenic *Escherichia coli* [3]. Estimated disease-associated annual costs of 2.4 billion Euro in the EU further underline the significant public health and socioeconomic burdens of campylobacteriosis [3]. The spiral-shaped and highly motile Gram-negative bacteria usually reside within the intestinal tract of warm-blooded vertebrates including avian livestock such as chicken and poultry as commensals and are acquired by humans via the food chain upon consumption of contaminated meat or surface water [4,5,6]. Following establishment within the intestinal tract, the pathogens attach to and invade the colonic epithelia cells, and subsequently induce pro-inflammatory innate and adaptive immune responses leading to immune cell infiltrates, crypt abscesses, and ulcerations [7,8,9]. Following an incubation period of 2–5 days, infected humans present with symptoms of varying degree that might mount in the symptom complex of acute campylobacteriosis characterized by abdominal cramps, watery or even bloody diarrheas with mucous discharge, and fever [10,11]. Whereas in the vast majority of cases patients require, if at, symptomatic therapy such as replacement of fluids and electrolytes, antimicrobial treatment might be indicated in individuals with immunosuppressive comorbidities [10,11]. Usually, the course of the disease is self-limited and symptoms resolve within two weeks, but post-infectious complications such as Guillain-Barré syndrome (GBS), reactive arthritis, and chronic inflammatory morbidities in the intestinal tract might occur in rare instances [9,10,11].

Recently, our group has established an acute in vivo *C. jejuni* infection model providing a reliable experimental tool to survey distinct molecules for their anti-pathogenic, anti-apoptotic, anti-inflammatory, and cell-regenerative properties during campylobacteriosis on a pharmaceutical level. Therefore, secondary abiotic interleukin (IL) 10^−/−^ mice have been generated by broad-spectrum antibiotic pre-treatment in order to overcome the physiological colonization resistance mediated by the distinct murine gut microbiota composition which protects mice even from high-dose *C. jejuni* infection [12,13,14]. Whereas conventional mice are approximately 10,000 times more resistant to both lipopolysaccharide (LPS) and LOS as compared to humans [15], IL-10 gene deficiency renders mice susceptible to these Toll-like receptor-4 (TLR-4) ligands [16]. In fact, within one week following peroral *C. jejuni* challenge, secondary abiotic IL-10^−/−^ mice suffer from wasting and bloody diarrhea during this acute and non-self-limiting course of enterocolitis which is due to *C. jejuni* LOS induced and TLR4 dependent immune responses within intestinal, extra-intestinal, and even systemic tissue sites [16,17]. Our very recent pre-clinical intervention trials applying this clinical enterocolitis model mimicking key features of human campylobacteriosis revealed that defined dietary molecules such as carvacrol (abundant in thyme oil and oregano, for instance), but also distinct vitamins including vitamin C and vitamin D could effectively alleviate *C. jejuni* induced disease due to their anti-apoptotic, cell-regenerative, and immune-modulatory (i.e., anti-inflammatory) properties [18,19,20].

More than 20 years ago, the octapeptide NAP (i.e., NAPVSIPQ) has been identified as biologically active fragment derived from the activity-dependent neuroprotective protein (ADNP) [21]. Human ADNP is primarily expressed in both, the central and peripheral nervous system, but can also be found in distinct compartments and cell populations of the immune system such as the spleen, macrophages, and leukocytes, respectively [21,22,23,24]. The neuroprotective properties of NAP have been proven in vitro and further confirmed in in vivo models for Alzheimer’s disease, stroke, fetal alcohol syndrome, neonatal hypoxia, cerebral palsy, and closed head injury among others [21,25,26,27,28,29,30]. Particularly the microtubules could be identified as the main target for NAP action [31], enlisting the microtubule associated protein tau to microtubules upon direct interaction with the microtubule end binding proteins, EB1 and EB2 [32,33]. Consequently, the cells can be protected from oxidative stress-induced apoptosis [34]. Besides anti-oxidative mode of actions, immune-modulatory (i.e., anti-inflammatory) effects have been proposed as mechanisms underlying the neuroprotective properties exerted by NAP [35]. Importantly, the NAP target, EB1 is one of the major proteins with increased expression following interferon-γ (IFN-γ)/LPS macrophage activation as part of the crucial impact of microtubules on the immune response [36].

Our recent in vivo studies revealed that the anti-inflammatory effects of exogenous NAP were not restricted to the nervous system but could also be observed in the intestinal tract given that intraperitoneal application of NAP could effectively alleviate subacute and even acute small intestinal inflammation in mice [37,38]. These NAP associated anti-inflammatory properties were further confirmed in an acute inflammation model affecting a different intestinal compartment and induced by a different inflammatory stimulus, namely in acute colitis upon oral dextran sulfate sodium (DSS) application to mice [39]. Given that information regarding potential health-beneficial effects of NAP in infectious intestinal morbidities are lacking to date, we assessed the anti-pathogenic, anti-apoptotic, cell-regenerative, and anti-inflammatory effects of the octapeptide in the intestinal tract (and beyond) following exogenous NAP application to secondary abiotic IL-10^−/−^ mice suffering from acute *C. jejuni* induced enterocolitis in frame of our present preclinical intervention study.

## 2. Materials and Methods

### 2.1. Ethics Statement

All animal experiments were conducted in accordance with the European Guidelines for animal welfare (2010/63/EU) following approval by the commission for animal experiments headed by the “Landesamt für Gesundheit und Soziales” (LaGeSo, Berlin; on July 15, 2019, registration number G0104/19). Twice a day, clinical conditions of mice were assessed.

### 2.2. Generation of Secondary Abiotic IL-10−/− Mice

Female and male IL-10^−/−^ mice (all in C57BL/10 background) were reared under specific pathogen free (SPF) conditions in the same unit of the Forschungseinrichtungen für Experimentelle Medizin (FEM, Charité-University Medicine Berlin). Mice were maintained in cages including filter tops within an experimental semi-barrier (accessible only with lab coat, overshoes, caps, and sterile gloves) under standard conditions (22–24 °C room temperature, 55 ± 15% humidity, 12 h light/12 h dark cycle) and had free access to autoclaved standard chow (food pellets: ssniff R/M-H, V1534-300, Sniff, Soest, Germany).

In order to deplete the gut microbiota, 3-week-old littermates were transferred into sterile cages (maximum of 4 animals per cage) and subjected to a broad-spectrum antibiotic treatment for eight weeks by adding ampicillin plus sulbactam (1 g/L; Ratiopharm, Ulm, Germany), vancomycin (500 mg/L; Cell Pharm, Hannover, Germany), ciprofloxacin (200 mg/L; Bayer Vital, Leverkusen, Germany), imipenem (250 mg/L; Fresenius Kabi, Bad Homburg, Germany) and metronidazole (1 g/L; B. Braun, Melsungen, Germany) to the drinking water (ad libitum) as described earlier [12,40]. Secondary abiotic IL-10^−/−^ mice were continuously kept and handled under strict aseptic conditions and received autoclaved food and drinking water in order to minimize the risk of contaminations.

### 2.3. C. jejuni Infection, Gastrointestinal Colonization, and Extra-Intestinal Translocation

For infection experiments, *C. jejuni* strain 81–176 that had initially been isolated from a diseased patient suffering from bloody diarrhea was freshly cultivated on Columbia agar (supplemented with 5% sheep blood) and Karmali agar plates (both from Oxoid, Wesel, Germany) after thawing from a stock. On two consecutive days (i.e., days 0 and 1), secondary abiotic IL-10^−/−^ mice were perorally challenged with 10^9^ colony forming units (CFU) in a volume of 0.3 mL phosphate buffered saline (PBS; Thermo Fisher Scientific, Waltham, MA, USA) by gavage [12]. In order to assess intestinal colonization properties *C. jejuni* loads were quantitatively surveyed in fecal samples over time post-infection (p.i.) and furthermore, in luminal samples derived from distinct parts of the gastrointestinal tract (i.e., from the stomach, duodenum, ileum, and colon) upon necropsy on day 6 post-infection (p.i.) by culture as stated elsewhere [12]. In brief, serial dilutions of samples were plated onto Columbia agar plates containing 5% sheep blood and Karmali agar plates (both from Oxoid, Wesel, Germany) and incubated in a jar under microaerophilic conditions for 48 h at 37 °C. For assessment of bacterial translocation to extra-intestinal compartments respective ex vivo biopsies were homogenized with a sterile pistil and serial dilutions cultivated accordingly. Furthermore, cardiac blood (0.2 mL) was immediately streaked onto Karmali agar plates.

### 2.4. Treatment with Synthetic NAP

The octapeptide NAP was synthesized as described earlier [41]. Starting on day 2 p.i. and lasting until the end of the experiment mice were either treated with synthetic NAP (1.0 mg per kg body weight; dissolved in NaCl 0.9%) or received vehicle (placebo) once daily via the intraperitoneal route. A potential antimicrobial effect of the solutions was excluded as reported earlier [38].

### 2.5. Clinical Assessment

Before and after pathogen infection, we quantitatively surveyed the clinical conditions of mice on a daily basis by using a standardized cumulative clinical score (maximum 12 points), addressing the clinical aspect/wasting (0: normal; 1: ruffled fur; 2: less locomotion; 3: isolation; 4: severely compromised locomotion, pre-final aspect), the abundance of blood in feces (0: no blood; 2: microscopic detection of blood by the Guajac method using Haemoccult, Beckman Coulter/PCD, Germany; 4: macroscopic blood visible), and stool consistency (0: formed feces; 2: pasty feces; 4: liquid feces) as described earlier [42]. Fecal blood positivity rates were assessed by the ratio of microscopically (including macroscopically) fecal blood positive mice and the total number of analyzed animals.

### 2.6. Sampling Procedures

On day 6 p.i., groups of mice were sacrificed by CO_2_ asphyxiation. Ex vivo biopsies from mesenteric lymph nodes (MLN), spleen, liver, kidneys, lungs, colon, and ileum as well as luminal gastrointestinal samples including stomach, duodenum, ileum, and colon were taken under sterile conditions. For serum cytokine measurements cardiac blood was taken. Large and small intestinal samples were collected from each mouse in parallel for microbiological, immunohistopathological, and immunological analyses.

### 2.7. Immunohistochemistry

In situ immunohistochemical analyses were done in large intestinal ex vivo biopsies following immediate fixation in 5% formalin and embedding in paraffin as recently reported [42,43]. In brief, in order to detect apoptotic epithelial cells, proliferation epithelial cells, macrophages/monocytes, T lymphocytes, regulatory T cells, and B lymphocytes, 5 µm thin colonic paraffin sections were stained with primary antibodies directed against cleaved caspase-3 (Asp175, Cell Signaling, Beverly, MA, USA, 1:200), Ki67 (TEC3, Dako, Glostrup, Denmark, 1:100), F4/80 (no. 14-4801, clone BM8, eBioscience, San Diego, CA, USA, 1:50), CD3 (no. N1580, Dako, 1:10), FOXP3 (clone FJK-165, no. 14-5773, eBioscience, 1:100), and B220 (no. 14-0452-81, eBioscience; 1:200), respectively. Positively stained cells were quantitated by a blinded independent investigator applying light microscopy (magnification 100× and 400×). The average number of respective positively stained cells in each sample was determined within at least six high power fields (HPF, 0.287 mm^2^, 400× magnification).

### 2.8. Pro-Inflammatory Cytokines in Intestinal, Extra-Intestinal, and Serum Samples

Distal large and small intestinal ex vivo biopsies were cut longitudinally, washed in PBS (Thermo Fisher Scientific) and strips of approximately 1 cm^2^ tissue as well as ex vivo biopsies derived from MLN (3 lymph nodes), liver (approximately 1 cm^3^), kidney (one half after longitudinal cut) and lung were placed into 24-flat-bottom well culture plates (Thermo Fisher Scientific) containing 500 µL serum-free RPMI 1640 medium (Thermo Fisher Scientific) supplemented with penicillin (100 U/mL) and streptomycin (100 µg/mL; Biochrom, Berlin, Germany). After 18 h at 37 °C, respective culture supernatants as well as serum samples were tested for interferon-γ (IFN-γ), tumor necrosis factor (TNF), monocyte chemoattractant protein-1 (MCP-1), and IL-6 by the mouse inflammation cytometric bead array (CBA; BD Biosciences, Germany) on a BD FACSCanto II flow cytometer (BD Biosciences). Nitric oxide concentrations were assessed by the Griess reaction [44].

### 2.9. Statistical Analysis

Medians and levels of significance were determined with GraphPad Prism v8, USA. The Student’s *t*-test was used for pairwise comparisons of normally distributed data, whereas the Mann–Whitney test was used for pairwise comparisons of not normally distributed data. For multiple comparisons, the one-sided ANOVA with Tukey post-correction was used for normally distributed data and the Kruskal–Wallis test with Dunn’s post-correction for not normally distributed data. Two-sided probability (*p*) values ≤ 0.05 were considered significant. Data were pooled from four independent experiments.

## 3. Results

### 3.1. Gastrointestinal Pathogen Loads Following NAP Treatment of C. jejuni Infected Secondary Abiotic IL-10^−^/^−^ Mice

**Figure 1:**
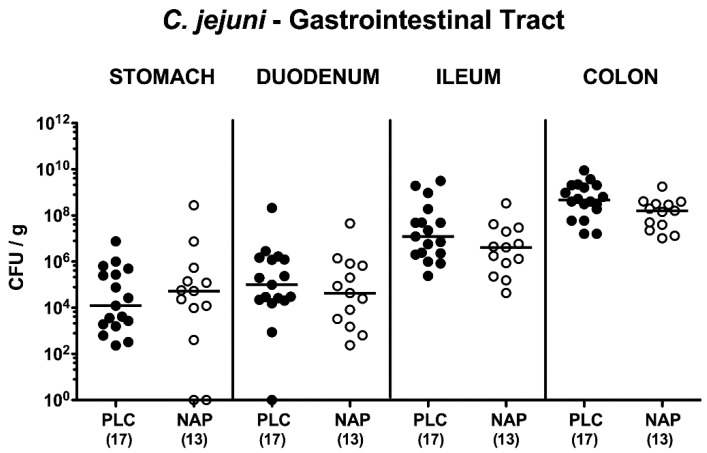
Gastrointestinal pathogen loads following NAP treatment of *C. jejuni* infected secondary abiotic IL10^−/−^ mice. On day (d) 0 and d1 secondary abiotic IL10^-/-^ mice were perorally infected with *C. jejuni* strain 81-176 by gavage and treated intraperitoneally with either synthetic NAP (open circles) or placebo (PLC; closed circles) from d2 until d5. Upon necropsy on day 6 post-infection, luminal bacterial loads were quantitatively assessed in distinct gastrointestinal compartments by culture (in colony forming units per g; CFU/g) as indicated. Medians (black bars) and numbers of analyzed animals (in parentheses) are given. Data were pooled from four independent experiments.

Secondary abiotic IL-10^−/−^ mice were perorally challenged with 10^9^ viable *C. jejuni* strain 81–176 on days 0 and 1 and intraperitoneally treated with synthetic NAP or placebo once daily from day 2 until day 5 p.i. Cultural analyses revealed that irrespective of the treatment regimen *C. jejuni* could stably establish within the murine intestines as indicated by median loads of 10^9^ bacterial cells per g feces (Figure S1). Upon necropsy on day 6 p.i., pathogen loads were comparable in distinct parts of the gastrointestinal tract such as stomach, duodenum, ileum and colon of NAP and placebo treated mice (n.s.; Figure 1). Hence, intraperitoneal NAP application did not affect colonization efficiencies of *C. jejuni* upon peroral infection of secondary abiotic IL-10^−/−^ mice.

### 3.2. Clinical Conditions Following NAP Treatment of C. jejuni Infected Secondary Abiotic IL10^−^/^−^ Mice

**Figure 2:**
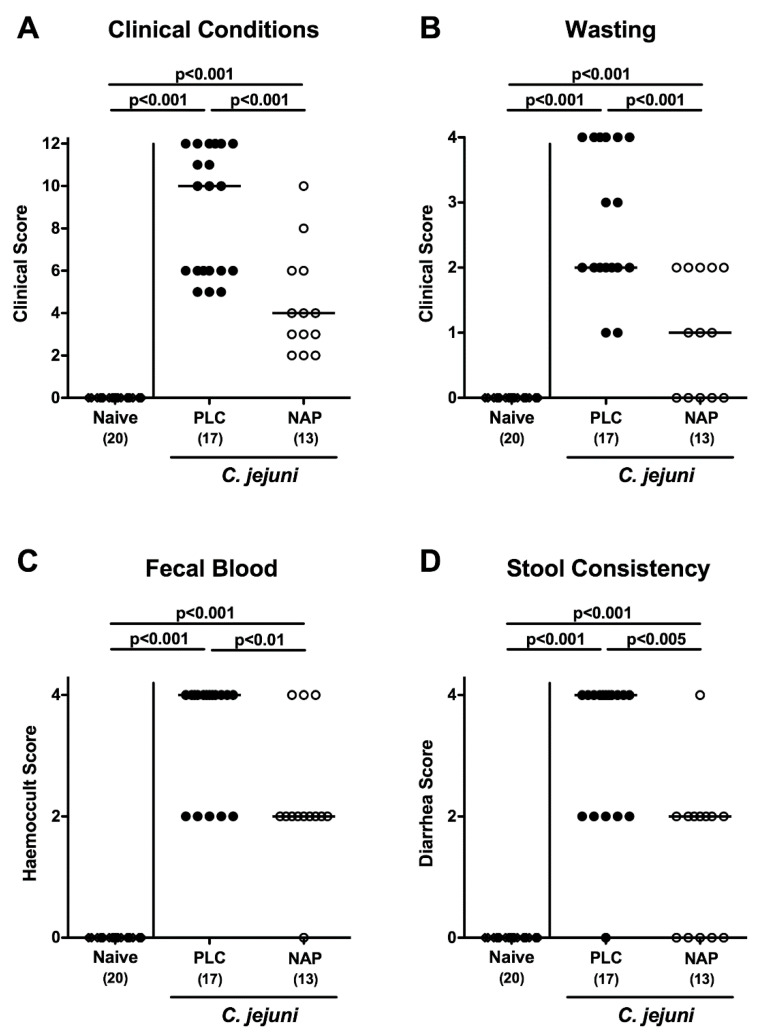
Clinical conditions following NAP treatment of *C. jejuni* infected secondary abiotic IL10^−/−^ mice. On day (d) 0 and d1 secondary abiotic IL10^−/−^ mice were perorally infected with *C. jejuni* strain 81–176 by gavage and treated intraperitoneally with either synthetic NAP (open circles) or placebo (PLC; closed circles) from d2 until d5. On d6 post-infection, (**A**) the overall clinical conditions, (**B**) wasting, (**C**) fecal blood, and (**D**) stool consistency were quantitatively assessed applying a standardized clinical scoring system. Naive mice served as negative controls (open diamonds). Medians (black bars), levels of significance (*p*-values) as determined by the Kruskal–Wallis test and Dunn’s post-correction and number of analyzed animals (in parentheses) are given. Data were pooled from four independent experiments.

In order to quantitatively survey the clinical effects of either treatment regimen over time we applied an established clinical scoring system [42]. As early as three days following the initial NAP application (i.e., day 5 p.i.), mice were less clinically compromised as compared to placebo controls (*p* < 0.05–0.001; Figure S2). On day 6 p.i., mice from the control group were suffering from severe campylobacteriosis, whereas NAP treated mice displayed lower clinical scores for overall clinical conditions and for wasting, abundance of fecal blood, and diarrhea in particular (*p* < 0.01–0.001; Figure 2). Hence, NAP treatment alleviated clinical signs of *C. jejuni* infection.

### 3.3. Colonic Epithelial Apoptotic and Proliferative Cell Responses following NAP Treatment of C. jejuni Infected Secondary Abiotic IL10^−^/^−^ Mice

**Figure 3:**
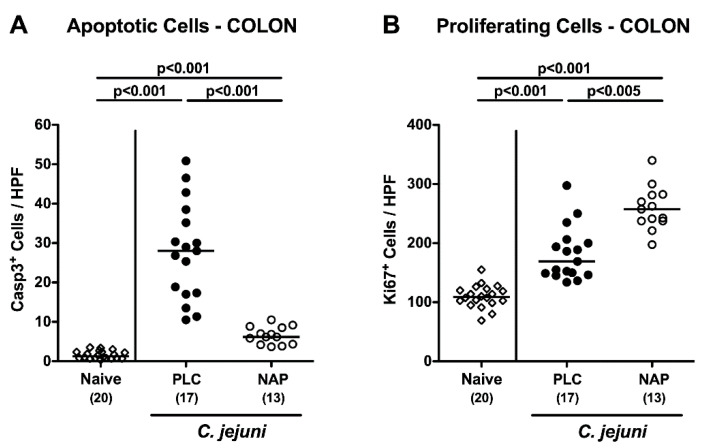
Colonic epithelial apoptotic and proliferative cell responses following NAP treatment of *C. jejuni* infected secondary abiotic IL10^−/−^ mice. On day (d) 0 and d1 secondary abiotic IL10^−/−^ mice were perorally infected with *C. jejuni* strain 81-176 by gavage and treated intraperitoneally with either synthetic NAP (open circles) or placebo (PLC; closed circles) from d2 until d5. On d6 post-infection, the average numbers of colonic epithelial (**A**) apoptotic (Casp3^+^) and (**B**) proliferating (Ki67^+^) cells were microscopically assessed in six high power fields (HPF, 400× magnification) per mouse in immunohistochemically stained colonic paraffin sections. Naive mice served as negative controls (open diamonds). Medians (black bars), levels of significance (*p*-values) as determined by the Kruskal–Wallis test and Dunn’s post-correction or the one-way ANOVA test and Tukey’s post-correction, as well as numbers of analyzed mice (in parentheses) are indicated. Data were pooled from four independent experiments.

Since apoptosis is considered as reliable parameter for the grading of intestinal inflammatory conditions [12], we enumerated the numbers of apoptotic cells in colonic epithelia following immunohistochemical staining of large intestinal paraffin sections with an antibody directed against cleaved caspase-3 (Casp3). Upon necropsy, increased numbers of Casp3^+^ colonic epithelial cells were assessed (*p* < 0.001), but with much lower counts in NAP as compared to placebo treated mice (*p* < 0.001; Figure 3A). Conversely, higher numbers of Ki67^+^ cells indicative for cell proliferation and regeneration could be determined in colonic epithelia of the NAP versus the control cohort on day 6 *p*.i. (*p* < 0.005; Figure 3B). Hence, NAP treatment resulted in less pronounced apoptotic cell responses in large intestines upon *C. jejuni* infection but promoted cell regenerative measures counteracting pathogen induced cell damage.

### 3.4. Colonic Innate and Adaptive Immune Cell Responses following NAP Treatment of C. jejuni Infected Secondary Abiotic IL10-/- Mice

**Figure 4:**
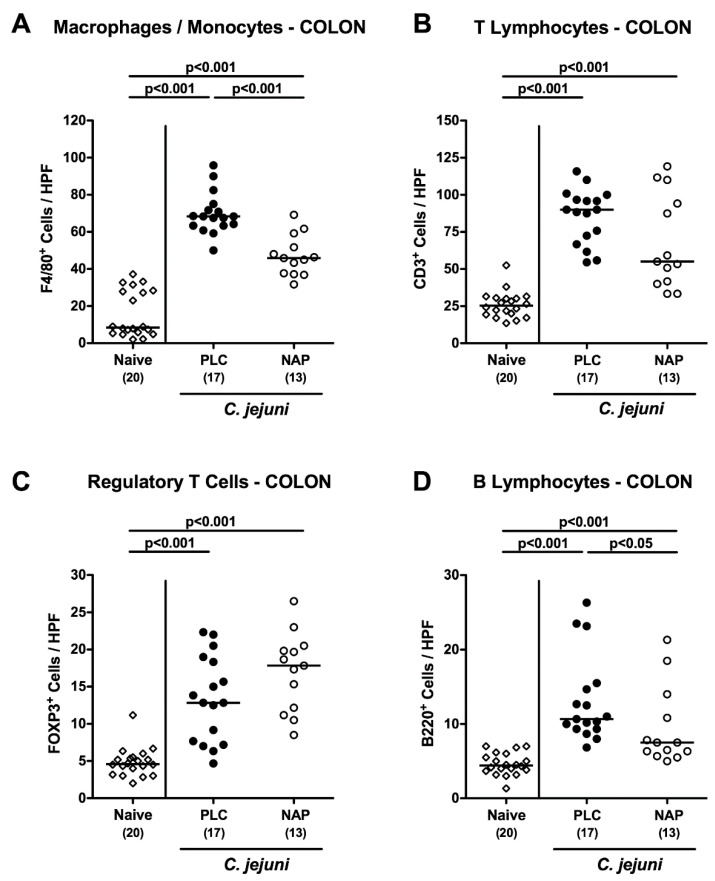
Colonic innate and adaptive immune cell responses following NAP treatment of *C. jejuni* infected secondary abiotic IL10^−/−^ mice. On day (d) 0 and d1 secondary abiotic IL10^−/−^ mice were perorally infected with *C. jejuni* strain 81-176 by gavage and treated intraperitoneally with either synthetic NAP (open circles) or placebo (PLC; closed circles) from d2 until d5. On d6 post-infection, the average numbers of (**A**) macrophages and monocytes (F4/80^+^), (**B**) T lymphocytes (CD3^+^), (**C**) regulatory T cells (FOXP3+), and (**D**) B lymphocytes (B220+) were microscopically assessed in six high power fields (HPF, 400× magnification) per mouse in immunohistochemically stained colonic paraffin sections. Naive mice served as negative controls (open diamonds). Medians (black bars), levels of significance (*p*-values) as determined by the one-way ANOVA test and Tukey’s post-correction or the Kruskal–Wallis test and Dunn’s post-correction, as well as numbers of analyzed mice (in parentheses) are indicated. Data were pooled from four independent experiments.

We next surveyed potential immune-modulatory properties of exogenous NAP during *C. jejuni* infection. Therefore, numbers of distinct innate and adaptive immune cell populations were determined in large intestinal paraffin sections applying in situ immunohistochemistry. *C. jejuni* infection was accompanied by increased abundances of innate immune cells such as F4/80+ macrophages and monocytes in the colonic mucosa and lamina propria (*p* < 0.001 vs. naive), but with lower counts in NAP as compared to placebo treated mice on day 6 p.i. (p < 0.001; Figure 4A). In addition, *C. jejuni* infected mice displayed higher colonic numbers of adaptive immune cell populations including CD3^+^ T lymphocytes, FOXP3^+^ regulatory T cells and B220^+^ B lymphocytes as compared to naive control animals (*p* < 0.001; Figure 4B–D). On day 6 p.i., B lymphocyte counts were lower in the colonic mucosa and lamina propria of NAP as compared to placebo treated mice, however (*p* < 0.05; Figure 4D). Notably, a trend towards lower large intestinal T lymphocytes and higher regulatory T cells could be observed following NAP versus placebo application but did not reach statistical significance due to high standard deviations in respective groups (n.s.; Figure 4B,C). Hence, exogenous NAP resulted in less distinct innate and adaptive immune responses upon *C. jejuni* infection.

### 3.5. Intestinal Pro-Inflammatory Mediator Secretion following NAP Treatment of C. jejuni Infected Secondary Abiotic IL10^−^/^−^ Mice

**Figure 5:**
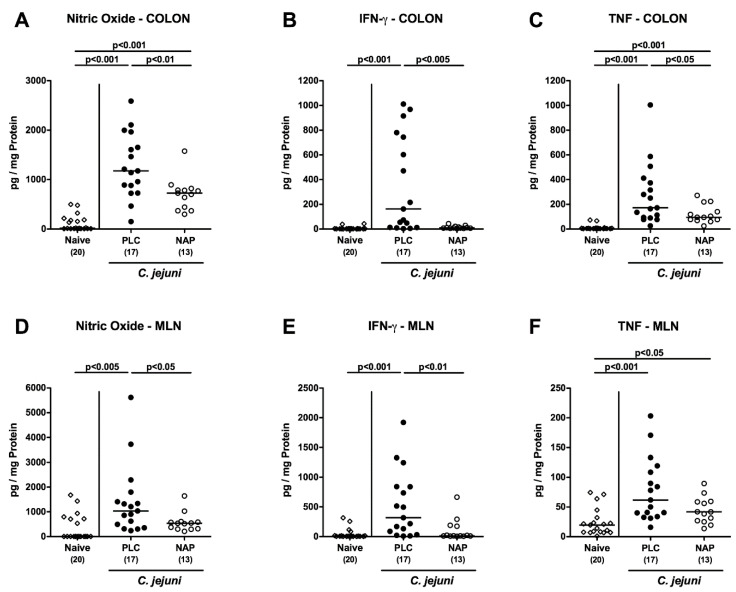
Intestinal pro-inflammatory mediator secretion following NAP treatment of *C. jejuni* infected secondary abiotic IL10^−/−^ mice. On day (d) 0 and d1 secondary abiotic IL10^−/−^ mice were perorally infected with *C. jejuni* strain 81–176 by gavage and treated intraperitoneally with either synthetic NAP (open circles) or placebo (PLC; closed circles) from d2 until d5. On d6 post-infection, nitric oxide (**A**,**D**), IFN-γ (**B**,**E**), and TNF (**C**,**F**) concentrations were measured in ex vivo biopsies taken from colon (**A**–**C**) and mesenteric lymph nodes (MLN; **D**–**F**). Naive mice served as negative controls (open diamonds). Medians (black bars), levels of significance (*p*-values) as determined by the Kruskal–Wallis test and Dunn’s post-correction, as well as numbers of analyzed mice (in parentheses) are indicated. Data were pooled from four independent experiments.

We next assessed pro-inflammatory mediator secretion in intestinal compartments following NAP treatment of *C. jejuni* infected mice. On day 6 p.i., increased nitric oxide and TNF concentrations could be measured in colonic ex vivo biopsies (*p* < 0.001; Figure 5A,C). These increases were, however, less pronounced in NAP as compared to placebo treated mice (*p* < 0.01 and *p* < 0.005, respectively; Figure 5A,C). Whereas colonic IFN-γ concentrations were multifold elevated in the placebo cohort on day 6 p.i. (*p* < 0.001), those measured in NAP treated mice did not differ from naive control animals (n.s.; Figure 5B). In MLN draining the inflamed intestines, less distinct nitric oxide and IFN-γ secretion was assessed upon NAP versus placebo treatment on day 6 p.i. (*p* < 0.05 and *p* < 0.01, respectively; Figure 5D,E), whereas a trend towards lower TNF concentrations was observed in the former versus the latter regimen (n.s.; Figure 5F). In addition, immune-modulatory properties of exogenous NAP were also effective in the distal small intestines as indicated by lower IFN-γ, TNF, MCP-1, and IL-6 concentrations in ex vivo biopsies obtained from the ileum of NAP versus placebo treated mice on day 6 p.i. (*p* < 0.05–0.005; Figure S3). Hence, exogenous NAP dampens *C. jejuni* induced pro-inflammatory mediator secretion in different parts of the intestinal tract.

### 3.6. Extra-Intestinal including Systemic Pro-Inflammatory Mediator Secretion following NAP Treatment of C. jejuni Infected Secondary Abiotic IL10^−^/^−^ Mice

**Figure 6:**
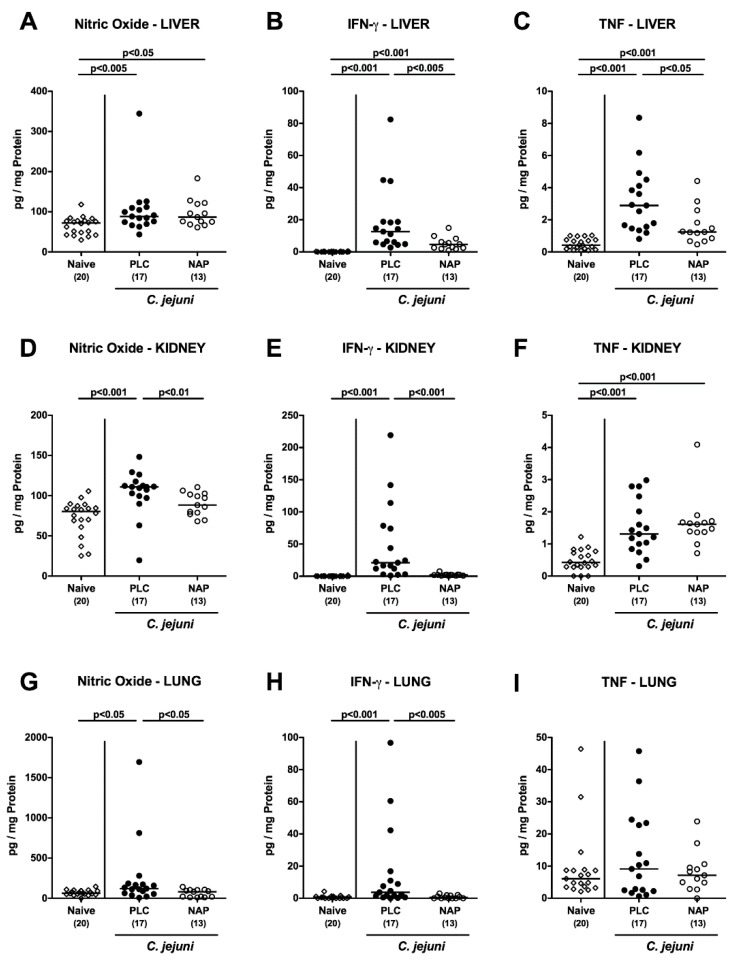
Extra-intestinal pro-inflammatory mediator secretion following NAP treatment of *C. jejuni* infected secondary abiotic IL10^−/−^ mice. On day (d) 0 and d1 secondary abiotic IL10^−/−^ mice were perorally infected with *C. jejuni* strain 81-176 by gavage and treated intraperitoneally with either synthetic NAP (open circles) or placebo (PLC; closed circles) from d2 until d5. On d6 post-infection, nitric oxide (**A**,**D**,**G**), IFN-γ (**B**,**E**,**H**), and TNF (**C**,**F**,**I**) concentrations were measured in ex vivo biopsies taken from liver (**A**–**C**), kidney (**D**-**F**), and lung (**G**–**I**). Naive mice served as negative controls (open diamonds). Medians (black bars), levels of significance (*p*-values) as determined by the Kruskal–Wallis test and Dunn’s post-correction, as well as numbers of analyzed mice (in parentheses) are indicated. Data were pooled from four independent experiments.

**Figure 7:**
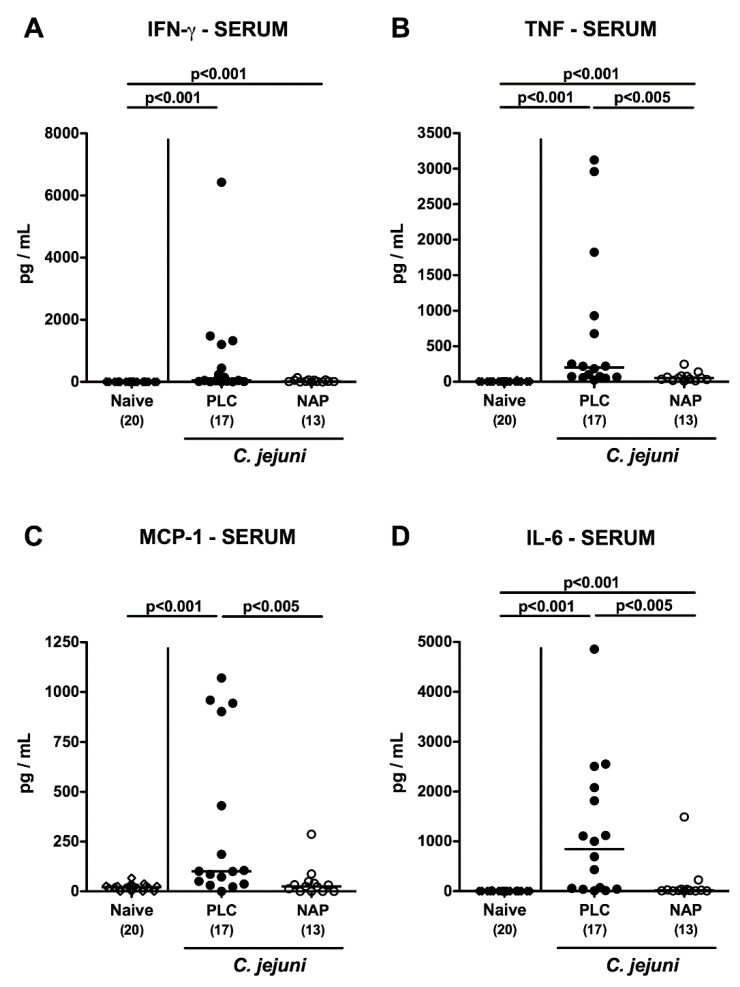
Systemic pro-inflammatory mediator secretion following NAP treatment of *C. jejuni* infected secondary abiotic IL10^−/−^ mice. On day (d) 0 and d1 secondary abiotic IL10^−/−^ mice were perorally infected with *C. jejuni* strain 81-176 by gavage and treated intraperitoneally with either synthetic NAP (open circles) or placebo (PLC; closed circles) from d2 until d5. On d6 post-infection, (**A**) IFN-γ, (**B**) TNF, (**C**) MCP-1, and (**D**) IL-6 concentrations were measured in serum samples. Naive mice served as negative controls (open diamonds). Medians (black bars) and levels of significance (*p*-values) as determined by the Kruskal–Wallis test and Dunn’s post-correction, as well as numbers of analyzed mice (in parentheses) are indicated. Data were pooled from four independent experiments.

We further addressed whether the inflammation-dampening effects of exogenous NAP were restricted to the intestinal tract or could also be observed in extra-intestinal compartments. Therefore, we measured pro-inflammatory mediators in ex vivo biopsies taken from liver, kidneys and lungs (Figure 6A–I). In fact, IFN-γ concentrations were lower in liver, kidneys and lungs of NAP as compared to placebo treated mice on day 6 p.i. (*p* < 0.005; Figure 6B,E,H), which also held true for nitric oxide concentrations measured in kidneys and lungs (*p* < 0.05–0.01; Figure 6D,G) and for hepatic TNF concentrations (*p* < 0.05; Figure 6C). Strikingly, the anti-inflammatory properties of NAP application were also effective systemically given that TNF, MCP-1, and IL-6 concentrations were all lower in serum samples taken from NAP versus placebo treated mice on day 6 p.i. (*p* < 0.005; Figure 7). Hence, NAP ameliorated *C. jejuni* induced pro-inflammatory immune response also in extra-intestinal—including systemic—compartments.

### 3.7. Bacterial Translocation following NAP Treatment of C. jejuni Infected Secondary Abiotic IL10-/- Mice

**Figure 8:**
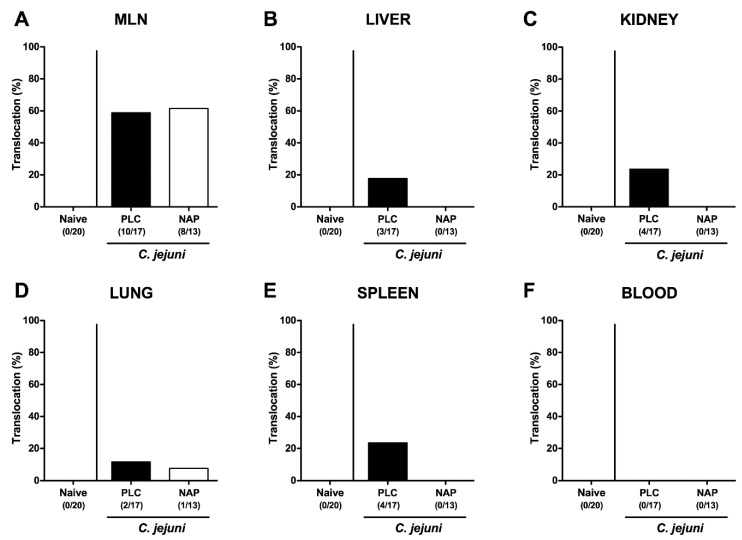
Bacterial translocation following NAP treatment of *C. jejuni* infected secondary abiotic IL10^−/−^ mice. On day (d) 0 and d1 secondary abiotic IL10^−/−^ mice were perorally infected with *C. jejuni* strain 81-176 by gavage and treated intraperitoneally with either synthetic NAP (open bars) or placebo (PLC; closed bars) from d2 until d5. On d6 post-infection, the abundance of *C. jejuni* was assessed in ex vivo biopsies derived from (**A**) mesenteric lymph nodes (MLN), (**B**) liver, (**C**) kidney, (**D**) lung, (**E**) spleen, and (**F**) cardiac blood by culture. Naive mice served as negative controls. The cumulative relative translocation rate of viable pathogens into the respective compartment out of four independent experiments (in %) as well as the numbers of culture-positive mice out of the total number of animals (in parentheses) are indicated.

We finally addressed whether viable bacteria were translocating from the infected intestines to extra-intestinal including systemic tissue sites of NAP treated mice. Whereas the pathogenic translocations rates to MLN of NAP and placebo treated mice were comparable on day 6 p.i. (58.8% and 61.5%, respectively; Figure 8A), *C. jejuni* could be cultured from liver, kidneys, lungs, and spleen of placebo control mice in 17.7%, 23.5%, 11.8%, and 23.5% of cases, respectively (Figure 8B–E), but less frequently in 0%, 0%, 7.7%, and 0% of respective compartments taken from NAP treated mice (Figure 8B–E). Of note, all cardiac blood samples were culture-negative for *C. jejuni* (Figure 8F). Hence, NAP treatment resulted in less frequent translocation of viable pathogens from the intestinal tract to extra-intestinal sites, including systemic compartments.

## 4. Discussion

Since antimicrobial resistance has become a major concern for public health in general and also *Campylobacter* become increasingly resistant to clinically important antibiotics [45], it is of utmost relevance to search for novel antibiotics-independent approaches to combat and/or even to prevent from campylobacteriosis. Our present preclinical intervention trial is the first study providing convincing evidence for disease-alleviating effects of the octapeptide NAP during acute colitis of enteropathogenic origin. Following intraperitoneal injections of synthetic NAP on four consecutive days secondary abiotic IL-10^−/−^ mice that had been perorally infected with *C. jejuni*, (i) harbored the pathogen in their gastrointestinal tract at comparable loads alike placebo control mice, but were (ii) clinically less compromised and (iii) displayed less distinct apoptotic, but (iv) enhanced cell proliferative and regenerative responses in colonic epithelia. Furthermore, following NAP as compared to placebo treatment, (v) lower numbers of both innate and adaptive immune cell subsets infiltrating the colonic mucosa and lamina propria could be assessed that were accompanied by (vi) less pronounced secretion of pro-inflammatory mediators in distinct intestinal, extra-intestinal, and strikingly, even systemic compartments; and finally, (vii) by less frequent translocation of viable pathogens from the intestinal tract to extra-intestinal tissue sites.

The result of uncompromised gastrointestinal colonization properties of the pathogen following synthetic NAP application was rather expected since our proceeding in vitro tests revealed that neither the verum nor the placebo working solutions exerted any antimicrobial effects [38]. Whereas placebo treated mice were suffering from severe campylobacteriosis, exogenous NAP resulted in an overall better clinical outcome and in less distinct wasting and bloody diarrhea in particular. In support, also in our previous studies applying different experimental model of intestinal inflammation, mice from the NAP treatment cohort had benefitted clinically and were suffering from less severe subacute ileitis following *Toxoplasma gondii* infection of mice with a human gut microbiota [37], which also held true for acute *T. gondii* induced ileitis [38] and acute DSS induced colitis [39] of conventional mice. The disease-alleviating effects of exogenous NAP were also effective on microscopic level given that mice were suffering from less severe *C. jejuni* induced colonic apoptosis, which was also the case when assessing numbers of cleaved caspase-3^+^ epithelial cell in the ileum and colon of mice suffering from acute *T. gondii* induced ileitis [38] or acute DSS colitis [39], respectively. In support, the prevention of caspase-3 activation and of subsequent apoptotic cell death highlighted the cell survival promoting features of NAP in the central nervous system [34,46]. Conversely, numbers of Ki67^+^ intestinal epithelial cells were increased following NAP treatment thereby promoting cell regenerative measures counteracting induced cell damaging responses as shown in our present and previous reports applying different acute intestinal inflammation models [38,39]. Interestingly, in a child suffering from the autistic ADNP syndrome (driven by heterozygous *ADNP* mutations) skin biopsies showed decreased Ki67^+^ staining, potentially associated with defective wound healing. Indeed, in a culture model of wound healing with cells derived from the mutated-*ADNP* skin biopsy, NAP showed protective effects [47]. In this respect, Adnp haploinsuffient mice (*Adnp^+/−^*) mimicked some of the skin deficient ADNP syndrome phenotype [47] and further showed distinct microbiota changes which were ameliorated by NAP treatment [48], potentially, through immune-modulatory effect. Given the gut microbiota changes towards higher loads of commensal bifidobacteria which are regarded as anti-inflammatory and probiotic bacterial species as observed in our previous NAP treatment studies applying mice harboring a murine or human gut microbiota [37,39,48], we will address the triangle relationship (“ménage à trois”) between *C. jejuni*, host commensal gut bacteria, and host immunity in future surveys.

Here, NAP exerted potent immune-modulatory effects since in the verum cohort far less innate as well as adaptive immune cells had accumulated in the colonic mucoasa and lamina propria of *C. jejuni* infected mice as compared to placebo controls. Furthermore, less pronounced secretion of pro-inflammatory mediators such as IFN-γ, TNF, IL-6, MCP-1, and nitric oxide (exerting oxidative stress to the intestinal epithelium) could be assessed in distinct intestinal compartments including the colon, ileum, and MLN draining the inflamed intestines of NAP versus placebo treated mice. In support, comparable NAP associated anti-inflammatory effects within the intestinal tract were observed in acute *T. gondii* induced ileitis and DSS colitis of mice [38,39]. Interestingly, monocytes, and T and B lymphocytes derived from peripheral blood mononuclear cells of healthy blood donors were shown to express ADNP [35]. Upon NAP treatment reduced T cell proliferation and activation could be observed in vitro which was accompanied by decreased secretion of pro-inflammatory cytokines including IFN-γ [35]. Furthermore, NAP effectively suppressed TNF and IL-6 secretion in murine macrophages in vitro [24] and of TNF in head trauma in vivo [27].

Remarkably, the inflammation-dampening effects of exogenous NAP were not restricted to the intestinal tract but could also be observed in extra-intestinal compartments as indicated by lower IFN-γ, TNF, and nitric oxide concentrations measured in liver, lung, and kidney ex vivo biopsies. Strikingly, the potent anti-inflammatory properties were also effective systemically given that IFN-γ, TNF, MCP-1, and IL-6 serum concentrations were lower upon NAP as compared to placebo treatment of *C. jejuni* infected mice. In support, in both, acute *T. gondii* ileitis and acute DSS colitis less pronounced extra-intestinal pro-inflammatory mediator secretion could be assessed following NAP as compared to placebo treatment [38,39] which also held true for systemic anti-inflammatory effects observed in acute murine ileitis [38].

Whereas ADNP is supposed to be a secreted protein [22,49,50,51], NAP is hypothesized to exhibit its systemic immune-modulatory functions in a cytokine-like (i.e., paracrine or apocrine) fashion [23].

Importantly, the NAP target, EB1 is one of the major proteins with increased expression following IFN-γ/LPS macrophage activation as part of the microtubules’ crucial impact on the immune response [36]. In this respect, macrophages display significant heterogeneity and plasticity. IFN-γ, LPS, and Bacillus Calmette-Guerin (BCG) induce the M1 phenotype, secreting a large number of pro-inflammatory cytokines (e.g., TNF, IL-6, IL-12), while transforming growth factor β (TGF-β) or glucocorticoids activate alternative pathways differentiating macrophages into the M2 phenotype, which exhibit increased secretion of IL-10, promote angiogenesis, wound healing, and regulate inflammatory responses [52]. Macrophage activation involves dramatic morphological changes in cell shape due to the rearrangement of actin and microtubules [53]. Furthermore, EB1/EB2 bind transient receptor potential cation channel subfamily M member 4 (Trpm4) possibly affecting T cell/Th1-Th2 activation, with Th1 secreting IFN-γ [54]. Given the ADNP/EB1/microtubules interactions, these findings indicate immune modulations by ADNP/NAP. Indeed, NAP inhibited LPS-induced secretion of TNF, IL-6, and IL-12 from ADNP-expressing macrophages [24] and increased the polymerized microtubules network area in neuronal-like cells [55]. Importantly and most relevant to the current study, NAP protected C57BL/6j mice that were perorally infected with *Toxoplasma gondii* and developed acute ileitis due to Th1-type immune responses [38].

Furthermore, here, translocation of viable pathogens from the infected intestines to extra-intestinal tissue sites such as liver, kidney, and spleen occurred in none of the NAP as opposed to the placebo treated *C. jejuni* infected mice indicative for a less compromised epithelial barrier function in the former versus the latter. Even though all cardiac blood samples remained culture negative for *C. jejuni* irrespective of the treatment cohort, one needs to take into consideration that distinct soluble *C. jejuni* cell wall constituents with immunogenic potency such as LOS among others might have circulated and contributed to the hyper-inflammatory scenario observed in placebo control mice.

The significant disease-alleviating effects upon NAP treatment are even more remarkable when considering that the synthetic octapeptide was administered only four times to mice. Hence, it is tempting to speculate that the effects would have even been more pronounced following increased doses, higher frequencies of application and start even before *C. jejuni* infection. The effects of such a prophylactic regimen will be addressed in future studies.

Our recent preclinical intervention studies further underlined the reliability of the here applied acute *C. jejuni* infection model in order to assess potential anti-pathogenic, anti-apoptotic, and immune-modulatory properties of promising candidate molecules in vivo. Secondary abiotic IL-10^-/-^ mice that had been pre-treated with vitamin C or carvacrol, for instance, harbored lower colonic pathogen loads and were suffering from less severe enterocolitis upon *C. jejuni* infection as compared to placebo control animals. Additionally, these treatment regimens dampened apoptotic epithelial and pro-inflammatory immune cell responses in the intestines that were accompanied by less pronounced pro-inflammatory cytokine secretion [19,20]. Potent immune-modulatory properties during acute *C. jejuni* induced enterocolitis could also be achieved by peroral application of vitamin D starting before *C. jejuni* infection [18].

In summary, we here show for the first time that exogenous NAP exerts potent disease-alleviating effects during acute murine enterocolitis caused by an enteropathogen such as *C. jejuni*. We conclude that synthetic NAP might be a promising novel option for treating acute campylobacteriosis in humans.

## Supplementary Materials

The following are available online at https://www.mdpi.com/2076-2607/8/6/802/s1, Figure S1: Kinetic survey of fecal pathogen shedding following NAP treatment of *C. jejuni* infected secondary abiotic IL10^−/−^ mice, Figure S2: Kinetic survey of clinical conditions following NAP treatment of *C. jejuni* infected secondary abiotic IL10^−/−^ mice, Figure S3: Ileal pro-inflammatory mediator secretion following NAP treatment of *C. jejuni* infected secondary abiotic IL10^−/−^ mice.

## Abbreviations

ADNP	Activity-dependent neuroprotective protein
BCG	Bacillus Calmette–Guerin
Casp3	Caspase-3
CBA	Cytometric Bead Array
CFU	Colony forming units
DSS	Dextran sulfate sodium
ECDC	European Centre for Disease Prevention and Control
EU	European Union
GBS	Guillain–Barré syndrome
HPF	High power fields
IFN-γ	Interferon-γ
IL	Interleukin
LOS	Lipooligosaccharide
LPS	Lipopolysaccharide
MCP-1	Monocyte chemoattractant protein-1
MLN	Mesenteric lymph nodes
PBS	Phosphate buffered saline
p.i	Post-infection
PLC	Placebo
SPF	Specific pathogen free
TGF-β	Transforming growth factor β
TLR-4	Toll-like receptor 4
TNF	Tumor necrosis factor
Trpm4	Transient receptor potential cation channel subfamily M member 4.
