# Supplementary figures and images for: Immune-modulatory Properties of the Octapeptide NAP in Campylobacter jejuni Infected Mice Suffering from Acute Enterocolitis
Source: Microorganisms. 2020 May 26;8(6):802. doi: 10.3390/microorganisms8060802 (PMC7356963; doi:10.3390/microorganisms8060802)

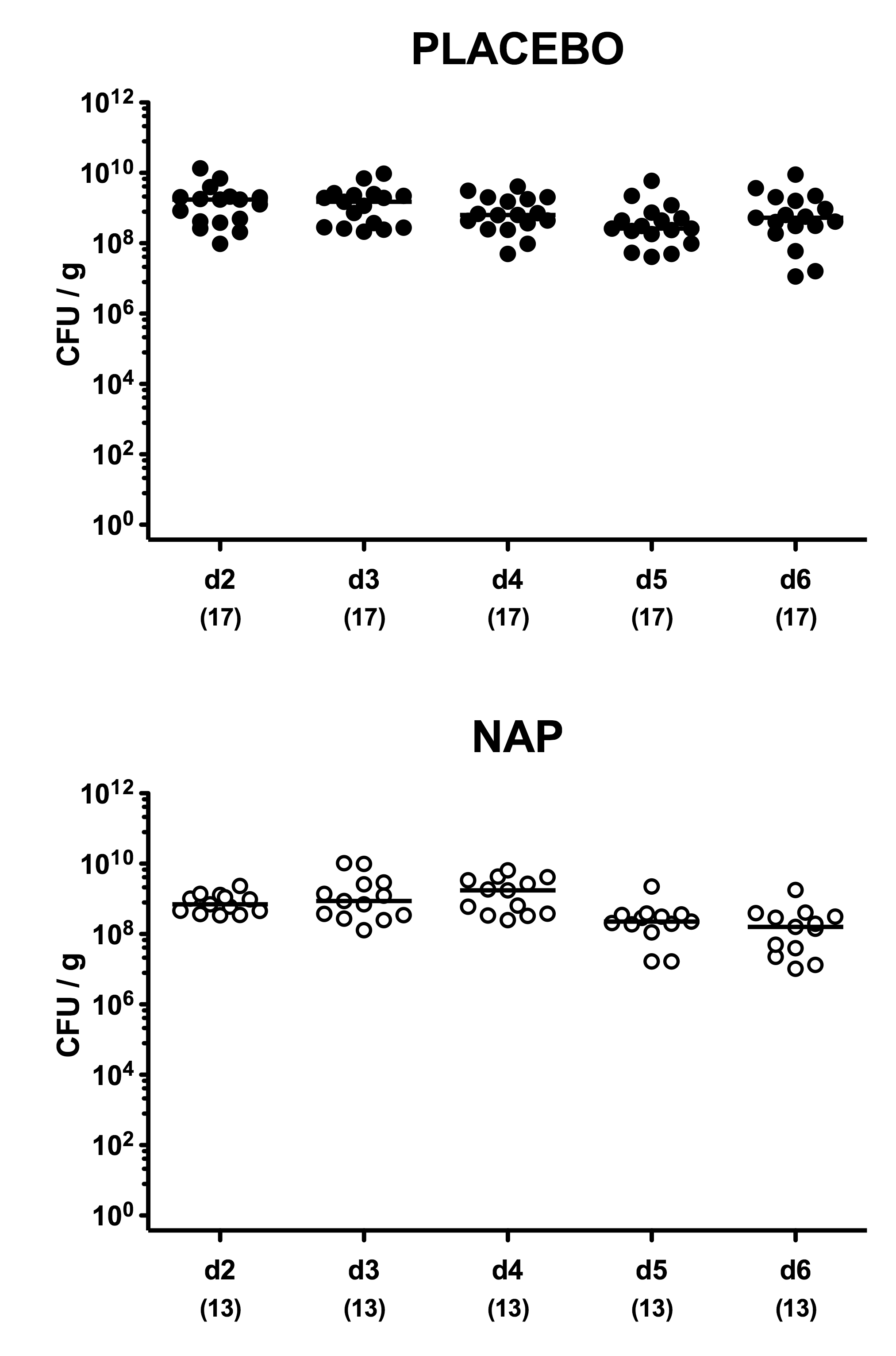

Supplement: Supplementary file 1 [file microorganisms-08-00802-s001.zip › FigS1_NAP_Campy_Kinetik_Group.tiff]

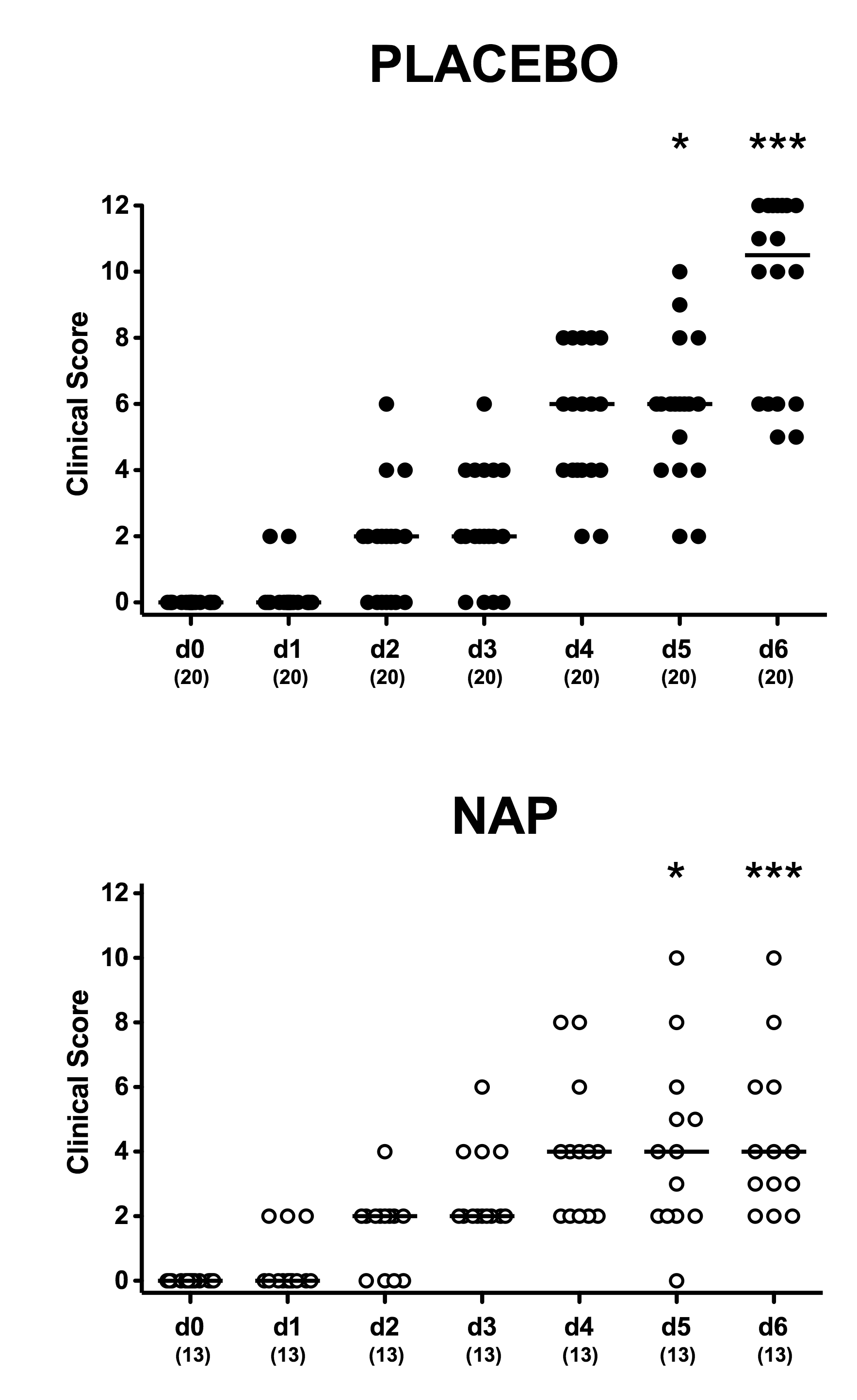

Supplement: Supplementary file 1 [file microorganisms-08-00802-s001.zip › FigS2_NAP_CS_Kinetik.tiff]

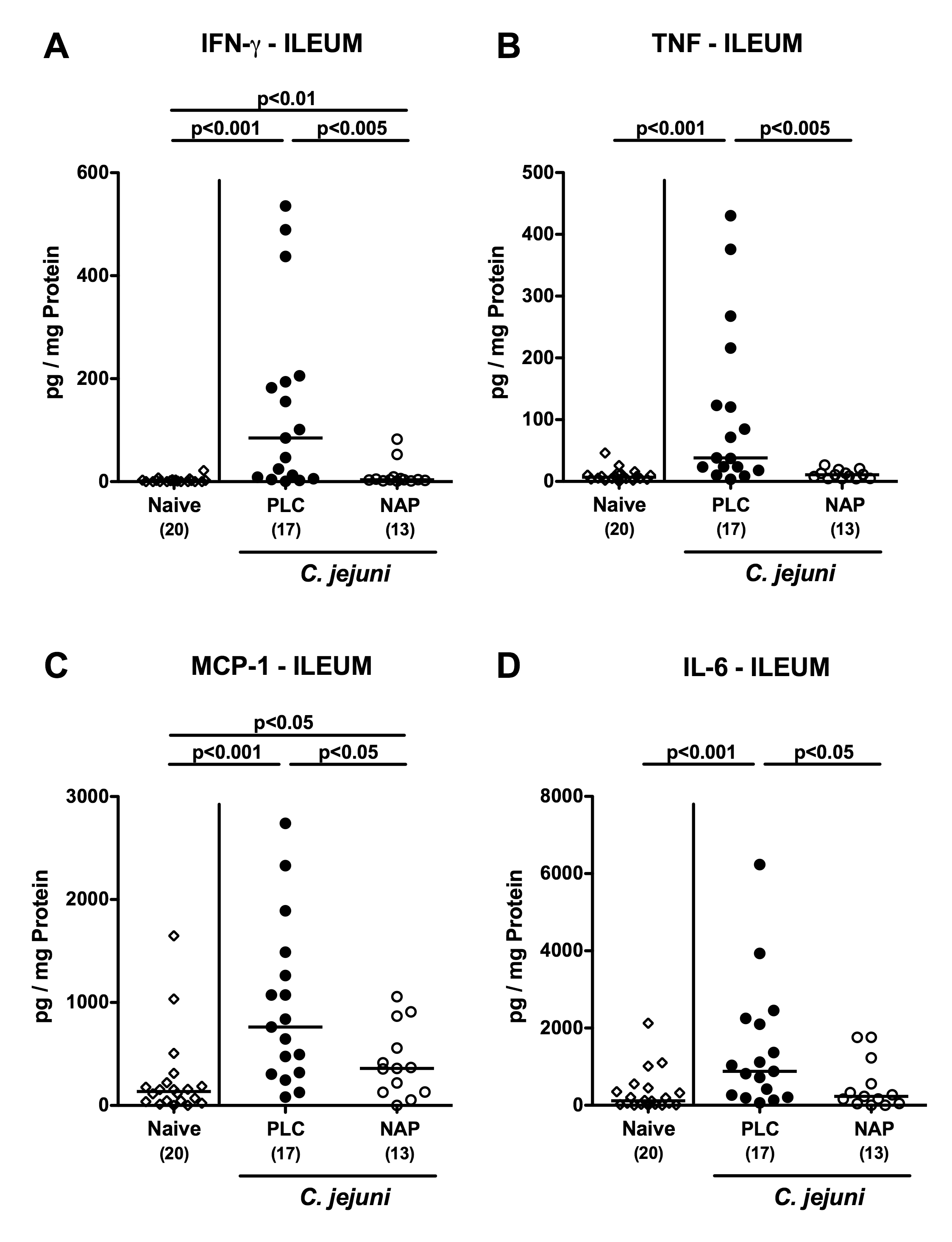

Supplement: Supplementary file 1 [file microorganisms-08-00802-s001.zip › FigS3_NAP_CBA_ILEUM.tiff]
